# CAR-T therapy toxicities: the importance of macrophages in their development and possible targets for their management

**DOI:** 10.1007/s12672-025-01776-0

**Published:** 2025-01-15

**Authors:** Joseph Norton, Patrick Stiff

**Affiliations:** 1 Internal Medicine Department, Division of Hematology, Oncology, and Transplant, University of Minnesota, 516 Delaware Street SE, PWB 14-100, Minneapolis, MN 55455 USA; 2 Internal Medicine Department, Division of Hematology-Oncology, Loyola University Medical Center, 2160 S 1St Ave, Maywood, IL 60153 USA

## Abstract

CAR-T cell therapies have risen to prominence over the last decade, and their indications are increasing with several products approved as early as second line in Large B Cell non-Hodgkin Lymphomas. Their major toxicities are the cytokine release syndrome (CRS) and the Immune-effector Cell Associated Neurotoxicity Syndrome (ICANS). These entities involve a hyperinflammatory cascade which is amplified through the mononuclear phagocytic system (MPS). Herein, we review the immune mediated adverse events related to CAR therapy, including their pathophysiologies, and current therapies. In particular, we discuss the emerging role of the MPS in both the toxicity and efficacy of CAR-T therapy, and possible avenues for the modulation of the MPS to optimize efficacy while minimizing toxicity.

## Introduction

Chimeric antigen receptor T cell (CAR-T) therapy is a treatment wherein usually autologous harvested T-cells, from patients with specific blood cancers are engineered to express a specified antigen. These are then infused into the patient where they recognize the desired antigen in vivo, and lead to cellular cytotoxicity for the antigen expressing cells [1–3]. CAR-T products have been developed for many malignancies but are most effective and lead to long term survival without disease in non-Hodgkin, B-cell lymphomas (NHL), acute lymphoblastic leukemia (ALL), CLL, and myeloma where they are now standard of care as early as second line [4–10]. However, CAR-T activation and expansion can cause severe inflammatory syndromes including the cytokine release syndrome (CRS) and the Immune-effector Cell Associated Neurotoxicity Syndrome (ICANS). These syndromes can be severe with Grade 3 or higher toxicity from some products as high as 28% and constitute a significant burden to CAR-T delivery as they often necessitate prolonged hospitalization or ICU level care [4–10]. Macrophages and the mononuclear phagocytic system (MPS) play an essential role in these inflammatory syndromes yet have heretofore been widely disregarded as a therapeutic target [11]. Herein we will discuss B-cell directed CAR-T products, the pathophysiology of CRS and ICANS, their current prophylaxis and treatment, as well as future directions for possible therapeutic advances. In particular, we will focus on what has been an overlooked avenue of therapy by our estimation: the macrophage.

## Current CAR-T products

The first FDA approval for a CAR-T product, tisagenlecleucel a CD19 CAR-T, was designed to treat relapsed or recurrent pediatric and young adult B-cell ALL in 2017 [4]. The field has expanded rapidly since that time. Four CD19 CAR-T products are now FDA approved for B-cell NHL, CLL and B-cell ALL [5–9], with three of them approved for use in the second line setting for relapsed or refractory Large B Cell NHL (LBCL) [5–7]. All these products are second generation CAR-T target CD-19 and incorporate a co-stimulator domain in its construction to enhance their activation and expansion in vivo [2, 4–7]. BCMA-directed CAR-T are also now available for use in multiple myeloma [10]. Currently commercially available CAR-T products utilize CD28 co-stimulation, such as axi-cel, and 4-1BB co-stimulation, such as liso-cel. Correlative data suggest that CD28 co-stimulation has a more rapid expansion kinetic, and thus these products have a higher CRS/ICANS side effect profile than does 4-1BB co-stimulated products [12]. It is unclear if this translates to improved efficacy, and both CD28 and 4-1BB costimulatory products are approved for use, even in the same indication as axi-cel and liso-cel, i.e. for relapsed and refractory LBCL [12, 13]. This review focuses on the toxicities of CAR-T cells for NHLs.

## The mononuclear phagocytic system

Important to understand along with T-cell cytotoxicity in CAR-T therapies is the MPS. Monocytes, macrophages, and dendritic cells make up a disbursed system, the MPS, which all derive from a common myeloid origin [14]. This system permeates all tissues and is integral to antigen presentation, innate immune responses, tissue repair, and many other tissue-specific roles [14–17]. This system replicates peripherally, especially in certain tissues such as the CNS and skin, as well as being replenished from blood derived monocytes [14, 15]. After complete ablation with etoposide, counts recover in blood over 4–7 days and in tissues in about 7–14 days with exceptions for tissue-specific macrophages, such as bone marrow macrophages and splenic marginal zone macrophages, which can take up to one month [14, 18, 19]. There are generally two distinct activated phenotypes of the MPS, the classically activated M1 and the non-classically activated M2 phenotypes. The M1, classically activated macrophages are pro-inflammatory and promote tissue damage. M1 cells express cytokines such as interleukin (IL) 1, IL-6, and tumor necrosis factor alpha (TNF-α). M1 activated cells typically dominate the early systemic response to infection, and in autoimmune inflammatory conditions such as rheumatoid arthritis. M2 activated cells in contrast are anti-inflammatory and profibrotic, expressing suppressive cytokines such as IL-10. M2 macrophages are often found in the tumor microenvironment of long-standing solid and liquid tumors, preventing immunosurveillance and immune cytotoxicity, as well as at sites of tissue repair and angiogenesis [20].

## Immune toxicities of CAR-T therapy

The most important side effects of CAR-T therapy are CRS and ICANS. While several definitions and grading schemas for CRS and ICANS have been proposed, the most commonly accepted and utilized ones come from the American Society of Transplantation and Cellular Therapy (ASTCT) which are summarized in Table 1.

**Table 1:** ASTCT CRS and ICANS Grading [12]

CRS parameter	Grade 1	Grade 2	Grade 3	Grade 4
Fever ≥ 38C	≥ 38C	≥ 38C	≥ 38C	≥ 38C
			With ≥ 1 of the Following	
Hypotension	None	Not requiring vasopressors	1 vasopressor	> 1 vasopressor
Hypoxia	None	< 6 L O2 by nasal cannula or equivalent	> 6 L O2 by nasal cannula or equivalent	Positive pressure ventilation

ICANS parameter	Grade 1	Grade 2	Grade 3	Grade 4
ICE score	7–9	3–6	0–2	0 (unable to perform ICE)
Depressed level of consciousness	Awakens spontaneously	Awakens to voice	Awakens only to tactile stimulus	Stupor or coma
Seizure	None	None	Any clinical seizure focal or generalized that resolves rapidly or non-convulsive seizures on EEG that resolve with intervention	Life-threatening prolonged seizure (> 5 min); or Repetitive seizures without return to baseline in between
Motor weakness	None	None	None	Deep focal motor weakness

Both of these entities are the product of immune therapy engaging immune effector cells, whether those are endogenous or infused. These toxicities are often progressive and can be severe to life threatening even if treatment is started. Fever is the initial hallmark of CRS, followed by hypotension, capillary leak with hypoxia, and due to these features, end organ damage. The pathophysiology of ICANS is similar to CRS but confined to the CNS with CNS specific symptoms rather than systemic ones. It does not require a preceding fever, and its symptoms are varied, ranging from tremors to seizures, cerebral edema, and coma [21]. Timing of these toxicities varies by product, disease characteristics, and prophylaxis used, but CRS typically occurs earlier, within the first week, whereas ICANS often occurs following an antecedent CRS [4–10]. CRS and ICANS both occur using other immune therapies as well, such as Bispecific T-Cell Engagers (BiTE)[22]; however we will focus on CAR-T here. The following are descriptions of the components of this pathophysiology.

### CRS pathophysiology

**Fig. 1:**
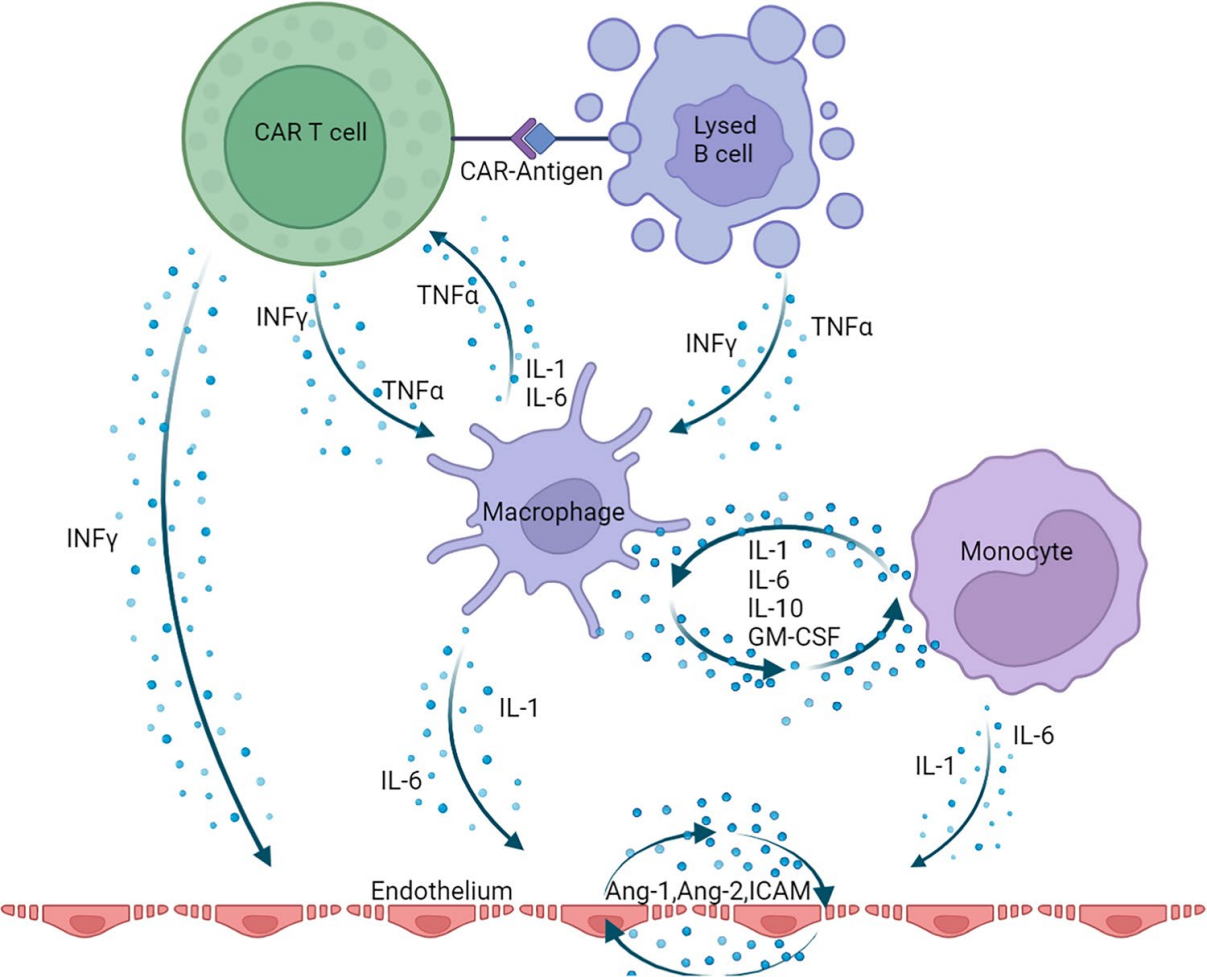
CRS Pathophysiology. Here CAR-T and the antigen connect causing target B-cell lysis. The resulting cytokines from both the CAR-T and target cell activate the macrophage. The macrophage and remaining MPS, here shown as a monocyte, create a positive feedback loop increasing the inflammatory cascade, and activate the endothelium increasing the lining’s permeability

CRS is a syndrome of systemic inflammatory response which bears a striking resemblance to Hemophagocytic Lymphohistiocytosis (HLH) and macrophage activation syndrome (MAS) where both inappropriate T-cell and macrophage activation causes a positive feedback loop of inflammation, visualized in Fig. 1 [23–25]. In CAR-T therapy, engineered T cells recognize the expressed antigen, are activated, and in turn activate the MPS. CAR-T cells activation and B-cell tumor lysis both result in release of TNF-α and interferon gamma (IFN-γ) [26, 27]. This initial inflammatory response activates the MPS which consequently releases TNF- α, IL-1, IL-6, and IL-10, creating a positive feedback loop through the MPS [11, 24, 27]. Further, these cytokines in turn recruit endothelial cells which activate and express Angiopoietin (Ang) 1 and 2, von Willebrand Factor (vWF), and intercellular adhesion molecule (ICAM) [24, 26]. Together, this creates the clinical picture. IL-1, IL-6, and TNF-α drive fevers while Ang1, Ang2, and ICAM loosen the otherwise tight vascular junctions leading to capillary leak, pulmonary edema, oxygen needs, and hypotension [24].

### ICANS pathophysiology

**Fig. 2.**
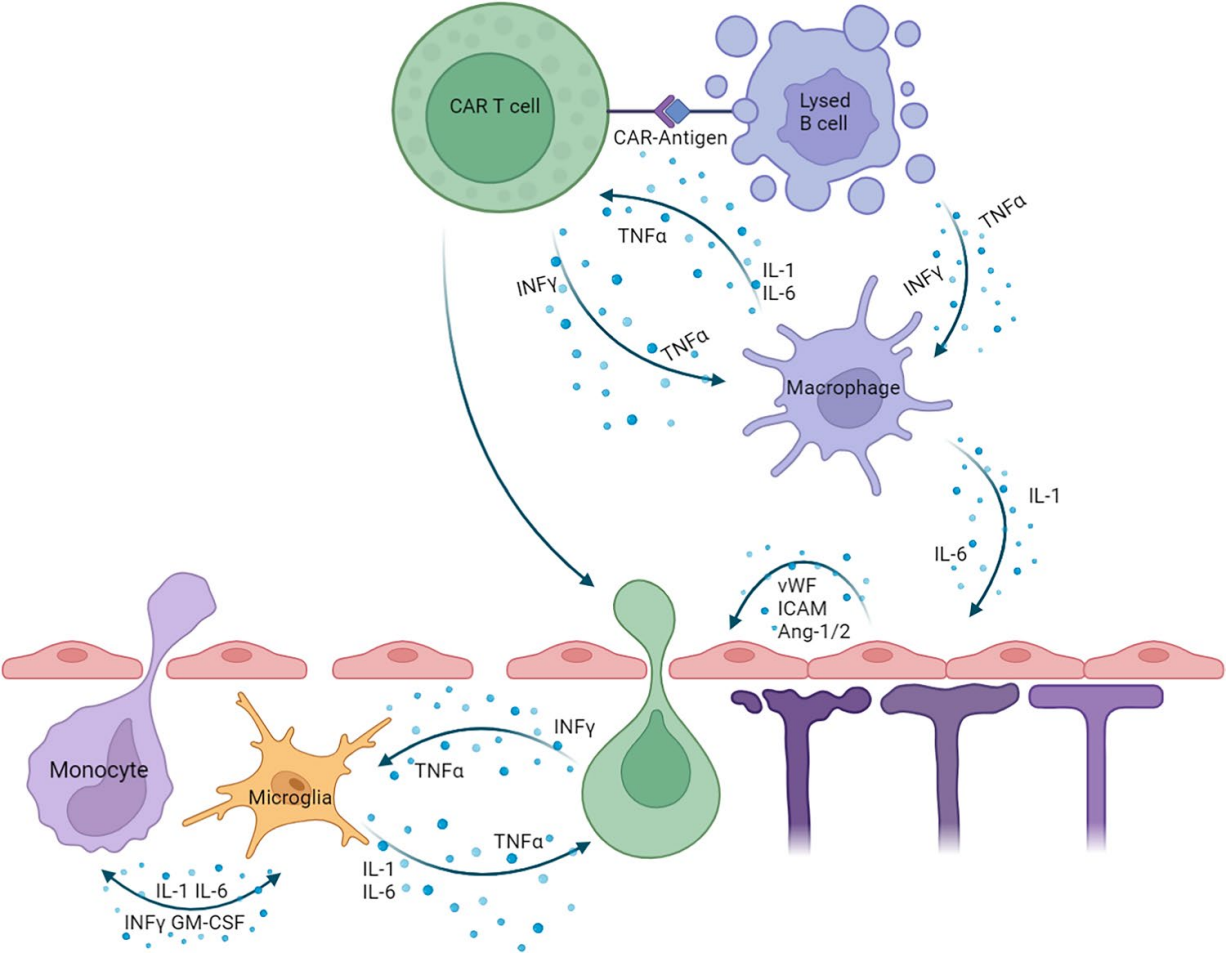
ICANS Pathophysiology. CAR-T cell and target B-cell signal the macrophage as in Fig. 1, but in ICANS the activation of endothelium causes weakening of the BBB, shown here with degenerating astrocyte foot processes, allowing for the entrance of Monocytes, and CAR-T into the CSF. This results in a localized positive feedback loop in the CNS

ICANS similarly develops from systemic hyperinflammation and is visualized in Fig. 2. However, patients who develop ICANS typically have greater elevations in IL-1, IL-6, IL-10, INF- γ, and Ang1/2 levels than their peers with only CRS, suggesting that a higher degree of inflammation is necessary to produce ICANS than CRS. This likely then explains the clinical experience given their frequent co-incidence in patients but with a typically lower incidence of ICANS [28, 29]. There are, however, distinct differences between CRS and ICANS. Most notably, endothelial activation is significantly greater in patients experiencing ICANS versus isolated CRS and may contribute to increased blood brain barrier (BBB) permeability with resultant cerebral edema and its clinical effects [28]. MRIs of patients with severe ICANS may show T2/Flair changes, signifying localized sites of cerebral edema, and sometimes contrast enhancement as evidence of the weakened BBB [24]. This BBB permeability permits the CAR-T cells and monocytes to cross over, causing the localized, CNS cytokine cascade. These newly entered cells perpetuate a positive feedback loop within the CNS leading to worsening ICANS with further increased BBB permeability, and greater CAR-T/MPS migration to the CNS [24, 30–33]. Correlative studies show that CSF from ICANS patients does contain the CAR product and a higher ratio of cytokines, such as IL-6, IFN-γ, and GM-CSF, than the blood supporting this hypothesis [24], and there is anecdotal evidence that intrathecal chemotherapy can mute this response.

### Immune effector cell HLH-like syndrome pathophysiology

HLH is a severe hyperinflammatory syndrome caused by T cell and macrophage activation unmitigated by the typical inhibitory natural killer (NK) cell function. Given that CRS and ICANS are foundationally similar in pathophysiology, caused by effector cell and macrophage activation, it is unsurprising that many initial CRS grading schemes included an HLH/MAS category [34]. However, there is a growing awareness that these entities should be differentiated, and in 2023 an ASTCT consensus statement regarding HLH-like syndrome after immune effector cell therapy proposed the terminology Immune effector cell HLH-like syndrome (IEC-HS) as well as diagnostic criteria and grading [34]. This is important for both clinical practice and research to ensure the same entity is discussed in each study, given their similarities. CRS/ICANS and IEC-HS share many features, such as fevers, hyperferritinemia, and hemophagocytosis, but they differ in key ways. Hallmarks of IEC-HS include progressive cytopenias, coagulopathy, and hepatic dysfunction whereas CRS centers on capillary leak, hypoxia, and hypotension instead. Their presentation timeline differ as well. CRS and ICANS overwhelmingly present early in the course of therapy, typically within 14 days, although this is variable based on the CAR product. IEC-HS, on the other hand, often presents several weeks to months after CAR product infusion and after an antecedent or ongoing CRS/ICANS process. To date all confirmed cases had such an antecedent CRS [34]. This category remains rare with only case reports at present, although this paucity of data may represent under-reporting this newly recognized toxicity [34]. As IEC-HS is a new category of secondary HLH, the particulars of its pathophysiology remain an active area of research.

## CAR-T and the MPS: toxicity

It must be noted that many of the cytokines and processes most implicated in CRS and ICANS have also been correlated with CAR-T engraftment and efficacy. Peak and area under the curve (AUC) numbers of CAR-T cells and associated expansion cytokines, such as IL-10, IL-15, and granzyme A, are associated with efficacy [5, 35, 36]. Expansion and persistence of the CAR-T population is necessary for long term efficacy and the use of lymphodepletion chemotherapy, such as fludarabine and cyclophosphamide, is used in order to remove the patient’s native pool “cytokine sinks,” from their native lymphocyte, so as to concentrate the effects of the cytokines on the CAR-T. However, peak and AUC levels of CAR-T and cytokines IL-8, IL-15, IL-6, IL-2, MCP1, GM-CSF, are also associated with higher rates and severity of toxicities [37, 38].

Therefore, determining which cytokines, and their source, that preferentially drive toxicity rather than efficacy is an area of ongoing research. Several such early studies have pointed to IL-1, IL-6, and MCP-1 as more predictive of toxicity rather than efficacy. It is important to note that these are produced by the myeloid MPS rather than the CAR T-cells [37, 38]. In fact, in murine models CAR products which are constructed to include an IL-1 antagonist or have IL-1/6 inhibition have significantly attenuated toxicity while maintaining efficacy [39]. Direct activation of the MPS via CAR-T directed to CD40L, a protein which couples to antigen presenting cells such as the MPS, caused increased rates of inflammatory toxicity in murine models further supporting their role in toxicity [39]. The inverse appears true as well. Depletion of the MPS prevented the inflammatory shock otherwise expected in a murine model utilizing zymogan [40]. This is also a logical premise based on data from other disease states, such as HLH/MAS. While CRS and ICANS are distinct entities from HLH/MAS, they share a similar driver of inflammation, a characteristic cytokine profile, and hemophagocytosis is present in many severe cases of CRS/ICANS [11, 24, 25, 40–43]. As such approaches targeting the MPS are appealing to prevent or ameliorate CRS/ICANS.

## CAR-T and the MPS: efficacy

While the MPS system is associated with toxicity, data is conflicting about the effects of the MPS on CAR efficacy. Some preclinical studies indicate the MPS are important effector cells in tumor clearance. Murine models have shown increased macrophages near tumors after CAR-T infusions, and correlative data in patients after liso-cel demonstrate increased tissue accumulation of macrophages compared to baseline in patients with durable response to therapy, further supporting the importance of the MPS in tumor clearance [31, 43]. Yet human trials in liso-cel patients have found that higher baseline levels of macrophage-derived INF in the local tumor tissue is correlated with lower rates of durable treatment response, suggesting elevated levels of macrophages, possibly immunosuppressive M2 macrophages, in tissue prior to CAR therapy could prevent CAR efficacy [41]. This suggests that patients may indeed benefit from transient MPS depletion shortly prior to CAR therapy to eliminate these M2 macrophages present, but thereafter allowing the inflammatory M1 macrophages to proliferate after CAR therapy given their potential efficacy post infusion.

The only time MPS depletion has been tested to reduce CAR-T toxicity was in the pivotal murine, leukemia model testing the MPS in CRS/ICANS using a bisphosphonate [11]. Bisphosphonates are utilized regularly in the rheumatology literature for MPS depletion [18, 45, 46]. They require endocytosis, making the process selective for the MPS, and cause apoptosis by disrupting ATP production [18, 45–47]. In the murine model of the MPS and CRS/ICANS, the authors showed that the addition of liposomal clodronate for three consecutive days prior to the CAR T infusion completely ablated the inflammatory toxicity noted in the control groups. However, it also decreased the maximum CAR T cell count in these mice and reduced the kinetics of leukemic clearance [11]. Multiple factors may be responsible for this outcome, such as dosing, which the study did not publish, and timing. The model dosed clodronate for three days immediately prior to CAR infusion, which provides the maximal duration and depth of MPS suppression. Another limitation of this study is the model itself. The leukemic cells of this model clear even in the control arm over the course of 14 days, and the MPS recovers over 4–7 days in the peripheral blood but in tissues often in 7–14 days [14, 18, 19]. Thus, a slower kinetic of CAR expansion and leukemic clearance following MPS recovery could not adequately be evaluated by this model. Taken together these data suggest that an administration of liposomal clodronate with time between depletion and CAR infusion may facilitate a slow return of the inflammatory M1 MPS to the system, allowing for adequate cytokine signaling and proliferation without being overwhelming, while removing immunosuppressive M2 MPS which prevent long-term efficacy. Further research into the specifics of MPS-CAR T cell efficacy and techniques for attenuating toxicity is warranted given these initial data.

## Possible future clinical MPS directed strategies

### Bisphosphonates

Bisphosphonates cause highly specific MPS apoptosis [18, 45–48]. However, as native compounds, such as oral alendronate, they are rapidly excreted in the urine, effective only in osseous tissue where they are actively absorbed [48]. To overcome this transient exposure in the soft tissues and blood, bisphosphonate exposure is increased if encased in multilaminar liposomes [49]. Liposomal clodronate is the most tested bisphosphonate for this purpose, but liposomal alendronate is occasionally used [18, 45, 49]. These drugs are well tested for this, having been utilized to explore MPS depletion in murine models of: colorectal cancer, myeloma, peritonitis, dengue fever, measles, RA, neuropathic pain, stroke, and post-PCI restenosis [19, 50–57]. As noted in the previous section, they were also utilized in establishing the role of the MPS in CRS/ICANS [11]. A novel pegylated form of liposomal alendronate has been tested as immunotherapy in mice as well and appears to enhance T-cell activity through ablating the tumor associated macrophages causing local immunosuppression [58]. Given their selectivity for the MPS and their demonstrated efficacy in these areas, they are a natural candidate for use in the CAR setting either during pre-infusion lymphodepletion as prophylaxis or as an abortive CRS/ICANS treatment.

However, despite the large body of research using liposomal bisphosphonates in mice, there are very few human trials to date. The BLAST and BLADE-PCI trials administered a liposomal alendronate at the time of percutaneous intervention to decrease rates of in-stent, arterial restenosis in humans, a process which has been found to be ablated in murine models presumably through monocyte depletion [59, 60]. These phase II trials used doses determined in an unpublished phase I trial, thus dose limiting toxicity is not available. The investigators used significantly lower liposomal alendronate doses (0.001 mg up to 0.08 mg total) than in the rabbit models of MPS depletion (3–6 mg/kg), in part since these studies were aimed to modulate rather than deplete the MPS [59–61]. They reported no increase in drug related adverse events as compared to placebo at these doses [59, 60]. Unfortunately the primary outcome of these phase II trials were negative, and thus the manufacturing company and the drug are no longer available.

Some challenges to liposomal drugs in humans do however exist, such as infusional toxicities, complement activation mediated pseudo-allergic reactions, accelerated blood clearance with repeated use, and vesicle instability [62]. However, many drugs have been developed to overcome these concerns such as liposomal doxorubicin and liposomal doxorubicin + cytosine arabinoside (CPX-351) [63, 64]. Perhaps the greatest unknown in this sphere is the preferential clearing of liver and spleen MPS seen in murine models of liposomal bisphosphonates [62]. Kupfer cells in the liver are vital to maintaining liver function and splenic macrophages have many tasks depending on subset, and thus how their clearance would affect blood tests and organ function is unknown and would require further testing [65, 66].

### Chemotherapy

One of the oldest mechanisms of MPS depletion is via etoposide, a DNA topoisomerase II inhibitor, which has demonstrated monocyte and T-cell cytotoxicity preference for over 40 years [67]. Further, murine models have shown that modulation and depletion of the MPS with etoposide decreases the inflammatory cytokine milieu and incidence of arthritis in such models [67–70]. It is also the drug of choice for HLH/MAS when cytotoxic chemotherapy is needed to treat patients refractory to steroids and cytokine therapy [71]. Thus, it is not surprising that etoposide has been recommended by some for use in severe, life threatening CRS where CAR-T destruction is considered an acceptable side effect of treatment [72]. One area where this has not been tested is in the lymphodepleting regimen prior to CAR T therapy. Modern lymphodepleting regimens derive from tumor infiltrating lymphocyte (TIL) studies, where prior chemotherapy benefited the infused TILs by decreasing T cells competing for pro-replication cytokines and removing suppressor cells from the milieu [73–75]. Current anti-CRS regimens do little to alter the MPS. Thus, further research into lymphodepleting regimens that add macrophage depletion is warranted, especially in groups at high risk of CRS such as patients with high tumor burden, high LDH, or high levels of CRP at the time of CAR-T therapy. However, exacerbating existing hematologic and infectious toxicities associated with lymphodepleting chemotherapy are potential drawbacks.

### Targeted approaches

Several different targets for the MPS system have been proposed and studied in murine and occasionally human models. Most of these studies have focused on the tumor microenvironment and shifting macrophages from M2 to M1 or eliminating the M2 population and their associated immunosuppressive T cells to enhance chemoimmunotherapy in solid malignancies [20, 76–81]. Many targets are under investigation for solid malignancies, including SIRPa, CD47, CSF-1R/CD115, the LILRB family of receptors, and the CD24-Siglec-10 axis among others [76–82]. Several of these have moved to human trials as well, notably a SIRP monoclonal antibody which is currently in phase 1 trials, and several LILRB targeted drugs including a CAR-T product [80, 83].

The diversity of targets each have their own benefits and draw backs, given how specific or non-specific they are. SIRPa is expressed both on macrophages and CD8 + T Cells which is excellent for the current trial in HLH, but would be devastating if T-cell preservation was required for CAR-T efficacy [77, 83]. Such a drug may act as an excellent lymphodepleting regimen however, eliminating both competing cytokine sinks in T-cells while removing endogenous M2 suppressor cells. LILRB family expression is confined to antigen presenting cells only, and LILRB4 is exclusive to the MPS [84]. As such targeting LILRB4 expressing cells for destruction, such as with a CAR-T product or antibody drug conjugate, would selectively deplete the MPS allowing for preservation of the CAR-T and treatment of established CRS/ICANS [80, 85].

## Current treatments for CRS and ICANS that inhibit the MPS

The current standard of care strategies for managing active CRS and ICANS also have an effect on the MPS. These have mainly relied on indirect alterations, such as targeting the cytokines produced by the MPS, rather than the direct strategies mentioned above.

### Glucocorticoids

Glucocorticoids remain the mainstay of treatment for more severe or persistent CRS or for the initial therapy of ICANS [21, 44]. The mechanism of action for glucocorticoids is multifaceted, but one of its major effects is the activation of the glucocorticoid receptor (GR), a DNA binding protein which regulates transcription initiation [86]. Activation of the GR in different tissues decreases vasodilatation and vascular permeability, decreases leukocyte adhesion and emigration to tissues, interferes with proinflammatory signaling through nuclear factor Kappa P (NF-κB), induces apoptosis of T-cells, suppresses MPS activity, and induces “tolerogenic” MPS cells [87–91]. Thus, for CRS/ICANS GCs work both by direct toxicity to the CAR T population, but also through decreasing inflammatory cytokine production, and creating a tolerogenic MPS environment. While low doses of GCs have not shown to affect the CAR T cell efficacy in human trials in both active disease and in prophylaxis, a retrospective analysis at MD Anderson of 100 patients undergoing CD-19 CAR T for LBCL showed that those who received > 186 mg of dexamethasone in total had shorter progression free and overall survival even after steroid discontinuation [92–94]. This may be due to the ongoing effects of the now tolerogenic MPS suppressing the CAR-T even after steroids have been withdrawn, or possibly disease burden at the time of therapy, a known risk factor for CRS and higher relapse rates.

### IL-6 blockade

The most commonly used therapy for the treatment of CRS is IL-6 blockade whose goal may also break or prevent the positive feedback loop through the MPS system [44]. This strategy completely prevented CRS in murine models when tocilizumab, a monoclonal antibody which competitively inhibits the IL-6 receptor was given at the time of CAR-T infusion [11]. The initial concern that interrupting the positive feedback loop in the MPS would hinder the efficacy of the CAR-T therapy appears unfounded, as studies have not shown IL-6 blockade affects CAR-T function in human or murine models and does not appear to affect disease prognosis after CAR-T therapy [11, 95, 96]. Thus, there are now protocols which use tocilizumab for prophylaxis as well [97]. IL-6 blockade with tocilizumab does not totally ablate ICANS in murine models, and this is true in clinical experience as well [11, 35–37]. Tocilizumab cannot cross the BBB, which may explain its failure to control the local, CNS hyperinflammatory cascade in ICANS [11, 44]. Further, as tocilizumab competitively inhibits the IL-6 receptor, there is the concern that its use may increase IL-6 concentration and make ICANS worse, which does appear to be true when tocilizumab is used in the prophylactic setting [6, 11, 24, 44, 97]. To overcome this, siltuximab, a humanized monoclonal antibody directed to IL-6 itself that can cross the BBB, has been tested in active disease and does show efficacy in both CRS and ICANS [98].

### IL-1 blockade

As with IL-6 blockade, the goal of IL-1 blockade therapy is to stop the positive feedback loop through the MPS. Anakinra is an IL-1 protein analog which competitively inhibits the IL-1 receptor and crosses the BBB [39, 99]. As with siltuximab in the IL-6 setting, anakinra has demonstrated efficacy in both the CRS and ICANS setting and has been used to significantly decrease CRS and ICANS if used prophylactically although further studies are warranted to ensure this does not compromise efficacy [100].

## Conclusions

CAR-T cell products are currently a mainstay of relapsed NHL and childhood/adolescent B-ALL treatment and encouraging data have been developed for relapsed multiple myeloma and adult B-ALL. As indications for these products increase, the need for ease of patient access and control of side effects likewise increases. The most important side effects of these therapies are CRS and ICANS which both are underpinned by a hyperinflammatory reaction which cannot be initiated without a positive feedback loop through the MPS. Our current treatment strategies modify this axis but indirectly. Despite current strategies, severe CRS and ICANS remain, and further treatment strategies remain an area of unmet need.

CAR-T therapies cause at times prolonged hospitalizations and in many cases prevent the cost-effective outpatient use of these agents given the high probability of hospitalization sometime in the first month after treatment. It is a natural next step to consider depletion or alteration of the mononuclear cell lineage directly to mitigate these costly implications. For prophylaxis, an agent which would affect a transient depletion or partial reduction of the MPS and T-cells during lymphodepletion would be ideal to allow a slower kinetic of CAR expansion, such as currently seen in 4-1BB using CAR products, while adequately depleting the T-cell “cytokine sink” prior to CAR infusion. For treatment of active disease, an MPS specific agent may be more effective than current options. MPS specific options include: chemotherapy, ADCs with different MPS target antigens, and bisphosphonates. These are briefly summarized in Table 2. Each has their own potential benefits and drawbacks. Directly affecting the MPS is a next logical step for the mitigation of severe adverse events in the use of CAR T products in hematologic malignancies.

**Table 2:** Possible future MPS directed strategies

Possible strategy	Affected target	Mechanism of specificity	Possible use
SIRPa	Macrophages and CD8 + T-Cells	Exclusive Cell Surface Expression	Lymphodepletion, CAR Ablating Rescue
Bisphosphonates	MPS	Phagocytosis Required for Effect	CAR Sparing CRS/ICANS Rescue
LILRB4	MPS	Exclusive Cell Surface Expression	CAR Sparing CRS/ICANS Rescue
Etoposide	Non-specific chemotherapy	More pronounced effect on T-cells and Macrophages	CAR Ablating Rescue, Lymphodepletion
Siglec-10	Predominantly Found on Cd4 + T-cells, B-cells, NK Cells, Monocytes	Cell Surface Expression	Lymphodepletion

## Data Availability

No datasets were generated or analysed during the current study.
